# Ultrasound‐guided percutaneous high‐frequency irreversible electroporation in porcine livers using four electrode needles: A feasibility and safety study

**DOI:** 10.1002/cam4.7035

**Published:** 2024-03-16

**Authors:** Chang‐Tian Li, Guo‐Dong Zhao, Wen‐Bo Zou, Zhen‐Hua Zhang, Yi Zhao, Rong Liu

**Affiliations:** ^1^ Faculty of Hepato‐Biliary‐Pancreatic Surgery The First Medical Center of Chinese People's Liberation Army General Hospital, Institute of Hepatobiliary Surgery of Chinese PLA Beijing China; ^2^ Department of General Surgery No. 924 Hospital of PLA Joint Logistic Support Force Guilin China; ^3^ REMD Medical Technology Shanghai China

**Keywords:** application, high‐frequency irreversible electroporation, liver, local ablation, porcine

## Abstract

**Background:**

Malignant liver tumors seriously endanger human health. Among different therapeutic approaches, high‐frequency irreversible electroporation (H‐FIRE) is a recently emerging tumor ablation technique. The objective of this study was to evaluate the feasibility and safety of ultrasound‐guided percutaneous H‐FIRE using four electrode needles in porcine livers.

**Methods:**

Twelve experimental pigs underwent percutaneous H‐FIRE ablation using a compound steep‐pulse therapeutic device. Liver tissues adjacent to the gallbladder, blood vessels, and bile ducts were selected as the ablation targets. Pigs were randomly divided into three groups: (1) immediately after ablation (*N* = 4), (2) 2 days after ablation (*N* = 4), and (3) 7 days after ablation (*N* = 4). Blood routine, liver and kidney function, and myocardial enzyme levels were measured before and after ablation. Ultrasound, contrast‐enhanced ultrasound (CEUS), contrast‐enhanced magnetic resonance imaging (MRI), and hematoxylin–eosin staining were performed to evaluate the ablation performance.

**Results:**

Ultrasound‐guided percutaneous H‐FIRE ablations using four electrode needles were successfully performed in all 12 experimental pigs. The general conditions of the pigs, including postoperative activities and feeding behaviors, were normal, with no significant changes compared with the preoperative conditions. The imaging features of ultrasound, CEUS, and MRI demonstrated no significant changes in the gallbladder walls, bile ducts, or blood vessels close to the ablation areas. Laboratory tests showed that liver function indices and myocardial enzymes increased temporarily after H‐FIRE ablation, but decreased to normal levels at 7 days after ablation. Histopathological examinations of porcine liver specimens showed that this technique could effectively ablate the target areas without damaging the surrounding or internal vascular systems and gallbladder.

**Conclusions:**

This study demonstrated the feasibility and safety of ultrasound‐guided percutaneous H‐FIRE ablation in porcine livers in vivo, and proposed a four‐needle method to optimize its clinical application.

## Graphical abstract

High‐frequency irreversible electroporation (H‐FIRE) ablation is a novel ablation technique for liver malignancies. It can reduce the destruction of extracellular matrix and maintain the potential of cell regeneration. However, in the treatment of liver malignancies, four‐needle electrodes are recommended in order to achieve complete ablation. Besides, it is superior to promote the application of ultrasound‐guided percutaneous H‐FIRE ablation in clinical practice. The objective of this study was to investigate the safety and efficacy of ultrasound‐guided percutaneous H‐FIRE ablation using four electrode needles in porcine livers in vivo. We believe that the findings of this study can optimize and simplify the clinical application of H‐FIRE ablation.

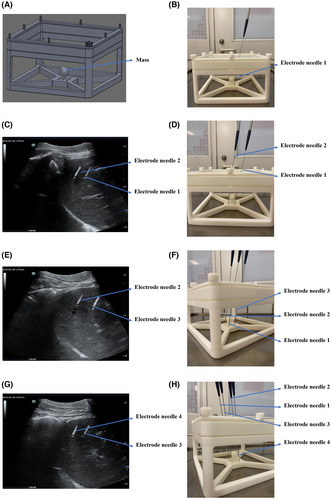

## 1 BACKGROUND

Image‐guided ablation is an effective standard treatment for patients with primary and metastatic liver tumors.^1^ Currently, thermal tissue destruction using microwave and radiofrequency ablation (MWA/RFA) is the most widely used ablation strategy, both of which are recommended to achieve curative effects in small and isolated liver malignancies.^2^, ^3^ However, traditional thermal ablation therapies have difficulties in achieving uniform temperatures and inevitably generate heat sink effects, which can lead to incomplete ablation of the tumor.^4^, ^5^, ^6^, ^7^ In addition, the heat generated during the ablation process may damage adjacent ductal structures and important organs, such as the gallbladder or intestinal tract.

Irreversible electroporation (IRE) is a novel alternative technique to thermal ablation in recent years that transmits ultrashort high‐voltage electrical pulses to the target through a fine probe to generate a strong external electric field to electroporate the cell membrane.^8^ The formation of nanopores in the cell membrane results in increased ion permeability across the membrane, leading to cell apoptosis due to the loss of physiological function of the membrane.^9^ It then activates the immune system to remove the cellular debris.^10^ Because IRE avoids the exposure of body tissues to extremely high temperatures, potential damage to the surrounding tissues can be minimized.^11^, ^12^, ^13^ It can reduce the destruction of the extracellular matrix, preserve the potential for cell regeneration, and create more predictable ablations in the relative absence of the “heat sink effect,” thus avoiding incomplete ablation caused by inhomogeneous heating.^14^, ^15^ Because of minor thermal effects in the ablation area, IRE can be performed close to the larger vessels and bile ducts without affecting the blood or bile flow.^16^ This enables re‐epithelialization and preservation of adjacent vascular and duct function after treatment.^17^, ^18^, ^19^ Owing to these advantages, IRE is now widely applied in solid tumor treatments, such as pancreatic cancer, prostate cancer, and renal cell carcinoma, and has proven to be an effective treatment option.^20^, ^21^

Despite the potential advantages of IRE over thermal ablation, some factors restrict its use. For instance, cardiac electrical asynchrony, with the potential for arrhythmia and severe tetanic muscle contraction, can cause serious intraoperative complications.^9^, ^22^ In addition, cell apoptosis in the ablation zone is relatively slow, which have affected may affect the ability of postoperative imaging to observe the extent of tumor destruction. Therefore, these factors have limited the use of IRE in the treatment of cancers for which resection or thermal ablation is unviable.

High‐frequency irreversible electroporation (H‐FIRE) has been developed as an alternative IRE approach to overcome many of the challenges of existing IRE. It uses ultrashort (1–2 μs) bipolar electrical pulses instead of conventional IRE using 50–100 μs monopolar electrical pulses to reduce the neuromuscular response and potentially avoid the need for intraoperative paralytic agents and cardiac synchronization.^23^, ^24^ High‐frequency irreversible electroporation ablation has been increasingly performed and has produced good results in prostate cancer.^20^, ^25^ However, the application of this technique in the liver, especially in areas near challenging locations,^26^ is worth further exploration. To create an effective electric field, the electrode needles must be parallel and 1.5–2.0 cm away from each other. Previous studies have demonstrated that open or CT‐guided H‐FIRE ablation using the dual‐needle method is feasible and safe in liver tissues. However, the cell necrosis area generated by the dual‐needle ablation method is spindle‐shaped, making it difficult to completely cover the liver tumors. Therefore, to achieve complete and precise ablation, the four‐needle ablation method are is recommended as an advantageous treatment strategy. In addition, ultrasound‐guided ablation has various advantages over CT guidance, such as real‐time monitoring and guidance, no radiation exposure, and relatively low cost and so forth. Therefore, the objective of this study was to investigate the feasibility and safety of ultrasound‐guided percutaneous H‐FIRE ablation in porcine livers in vivo, and to propose a four‐needle method to optimize its clinical application.

## 2 METHODS

### 2.1 Preoperative preparation

Twelve experimental pigs were included in this study. They were maintained for approximately 1 week to adapt to the environment and were randomly divided into three groups according to the follow‐up time points: (1) immediately after ablation (*N* = 4), (2) 2 days after ablation (*N* = 4), and (3) 7 days after ablation (*N* = 4). During breeding, all pigs were physically examined by professionals at the experimental center to ensure their suitability. All experimental pigs were observed twice a day on weekdays, and at least once a day on weekends or holidays. Before H‐FIRE ablation, all experimental pigs were fasted from solids and liquids for 12 h. We chose the right ear margin vein as the puncture point to establish venous access and then extracted 5‐mL venous blood tubes for blood routine, liver, and kidney function, and myocardial enzyme tests. After induction of general anesthesia and intubation, the pigs were placed in the supine position, and preoperative skin preparation was performed. After surgical site disinfection with iodophor, electrocardiogram electrodes were attached to each pig and pulse oximeter/carbon dioxide recorder sensors were placed appropriately in the mouth. This study was approved by the Animal Research and Ethics Committee of Chinese PLA General Hospital (S2021‐407).

### 2.2 Operative procedures

Ultrasound‐guided Percutaneous H‐FIRE ablation was performed using a Mindray I9 ultrasound system with a 3.5‐MHz convex probe, compound steep‐pulse therapeutic device (REMD Medical Technology, Shanghai, China), and matched electrode needles. Uniform ablation parameters were set in this study: pulse width of 10 μs, 150 pulses in a 1500 v/cm pulse train, post pulse width of 50 μs, and 100 pulses in a 1000 v/cm pulse train. The diameter of electrode needle is 1 mm, exposed length of the electrode needle is 1 cm, and the material of the electrode needle was made of austenitic stainless steel. The distance between the two adjacent electrodes is set to 1.5–2.0 cm, which is considered as effective distance.

Liver tissues adjacent to the bile ducts, blood vessels, and gallbladder were chosen as the target ablation areas. In addition, during the ablation process, ultrasound was used to monitor the changes in the ablation areas and ensure the positions and depths of the four electrode needles. In this study, ultrasound examinations were performed by the same doctors with >12 years of experience in abdominal ultrasound diagnosis.

#### 2.2.1 Step 1

**FIGURE 1:**
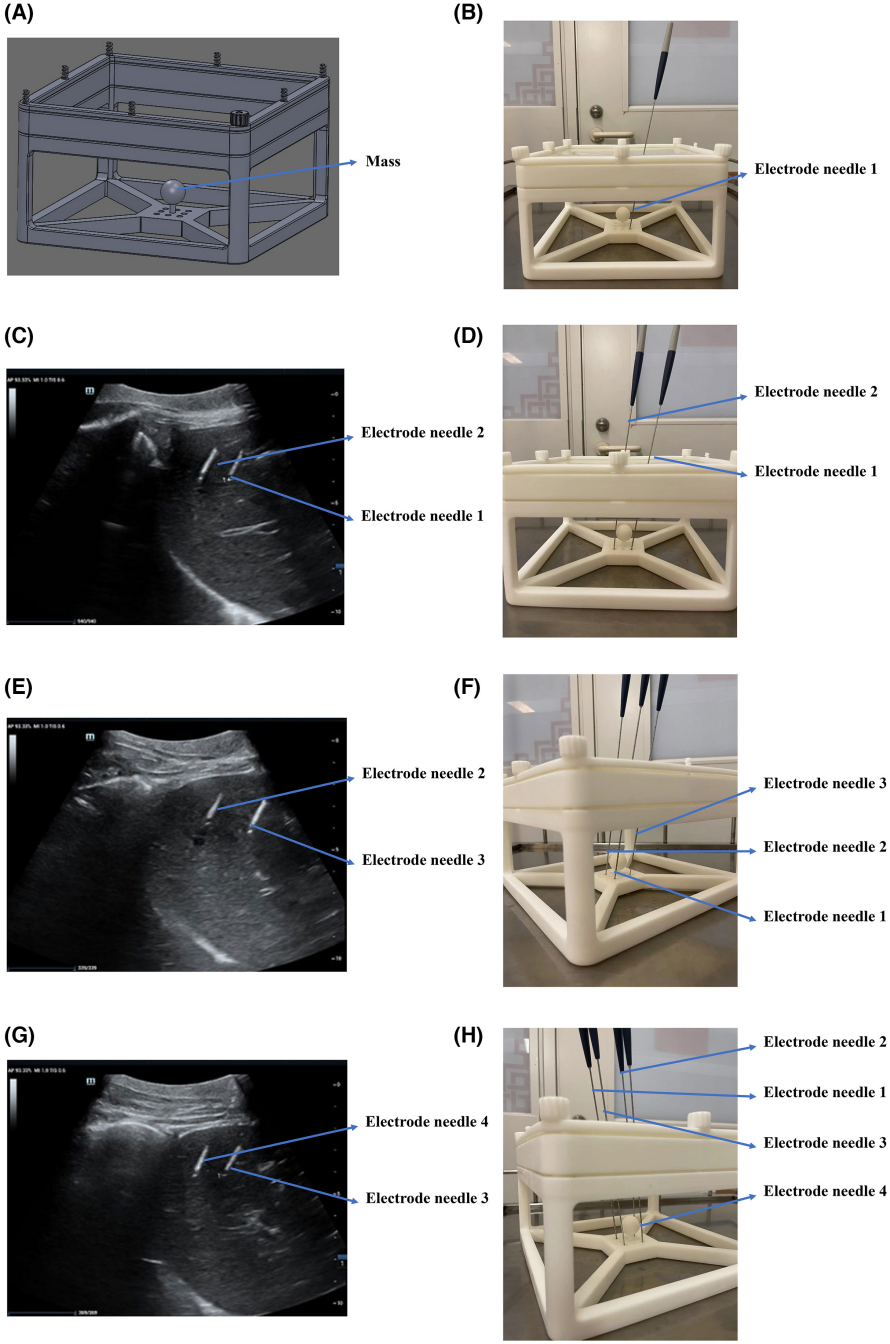
(A) Ultrasound‐guided percutaneous H‐FIRE ablation model using four electrode needles. The ball at the bottom simulates the mass of the liver. (B) The first electrode needle was inserted, and the angle and depth of the needle needed to wrap around the outer edge of the mass and leave a safety boundary of ≥0.5 cm. (C) Ultrasound was used to guide the insertion of the second electrode needle, which needed to be parallel to the first electrode needle. (D) Model of the arrangement of the first two electrode needles. (E) Ultrasound was used to guide the insertion of the third electrode needle, and which needed to be parallel to the second electrode needle. (F) Model of arrangement of electrode needles 1, 2, and 3. (G) Ultrasound was used to guide the insertion of the fourth electrode needle, which was parallel to the third electrode needle. (H) Model of the arrangement of four electrode needles.

First, the first electrode needle was inserted, and the angle and depth of the needle needed to wrap around the outer edge of the target domain and left a safety boundary of ≥0.5 cm (Figure 1A,B).

#### 2.2.2 Step 2

Next, the second electrode needle was inserted, and it needed to be parallel to the first electrode needle. The distance between these two electrode needles was about 1.5–2.0 cm (Figure 1C,D).

#### 2.2.3 Step 3

Then, the third electrode needle was inserted, and it needed to be parallel to the second electrode needle. The distance between these two electrode needles was about 1.5–2.0 cm (Figure 1E,F).

#### 2.2.4 Step 4

Finally, the fourth electrode needle was inserted, and it needed to be parallel to the third electrode needle. The distance between these two electrode needles was approximately 1.5–2.0 cm (Figure 1G,H). Therefore, all four electrode needles were parallel and 1.5–2.0 cm away from each other. Furthermore, electrode needles 2, 3, and 4 were placed at the same depth and position as electrode needle 1 to ensure that they could wrap around the outer edge of the target domain, leaving a safe boundary of ≥0.5 cm.

### 2.3 Postoperative management

Ultrasound, color Doppler flow imaging (CDFI), and contrast‐enhanced ultrasound (CEUS) examinations were performed immediately after H‐FIRE ablation. The low‐MI contrast mode was used during the CEUS examinations; the transmission power was 20%–40%, and the focus position was set around the ablation area. Then, 25 mg of the ultrasound contrast agent, sulfur hexafluoride microbubbles (SonoVue, Bracco Imaging), was dissolved in 5 mL of 0.9% normal saline to prepare a suspension, and each pig was given 2.5 mL of the SonoVue suspension followed by a bolus of 10 mL of normal saline through the ear margin vein. The timer on the ultrasound instrument was started when the injection was completed and the acquisition time of the CEUS data was set to 120 s.

### 2.4 Imaging follow‐up

The blood routine, liver and kidney function, and myocardial enzyme levels were measured at each follow‐up time point after H‐FIRE ablation. To evaluate the therapeutic effects of this technique, CEUS was performed immediately and 2 and 7 days after H‐FIRE ablation, and contrast‐enhanced magnetic resonance imaging (MRI) was performed 2 and 7 days after H‐FIRE ablation. The following ultrasound imaging features were assessed using echogenicity/signal intensity and the boundary and enhancement patterns of the ablation areas. MRI was performed using a 3‐T MRI system (TIM Trio, Siemens Healthcare). All MRI images were transferred to a picture archiving and communication system (PACS) (Centricity, GE Healthcare) and reviewed using the PACS workstation by the same radiologist with more than 10 years of experience.

### 2.5 Histopathological analysis

All experimental pigs were sacrificed at different follow‐up points, and the specimens were collected for histopathological analysis. Liver specimens were sliced for hematoxylin and eosin (H&E) staining. The samples were fixed in 4% paraformaldehyde at room temperature for 24 h, paraffin‐embedded, and cut into sections 4‐μm thick. The sections were dewaxed, hydrated, and stained with hematoxylin and eosin for 5 and 1 min, respectively. The ST Infinity H&E Staining System (Leica Biosystems, USA) was used at room temperature for histopathological examinations. The sections were then embedded and five random fields of view were obtained using an Olympus DP72 camera and Olympus image analysis software.

### 2.6 Statistical analysis

Continuous variables were expressed as mean (SD) or median (interquartile range) and were tested using Student's *t*‐test or Mann–Whitney *U* test. Categorical variables are reported as numbers with percentages and were tested using the chi‐squared test or Fisher's exact test. Two‐tailed *p* < 0.05 were considered significant for all statistical tests. GraphPad Prism (Version 8.0.1) and its corresponding resource package and SPSS 26.0 statistical software (IBM Corp, USA) were used for all statistical analyses and graphics.

## 3 RESULTS

### 3.1 Perioperative and laboratory test outcomes

Technical success was achieved in all 12 pigs. No muscle relaxants were used during the H‐FIRE procedures. Although muscle contractions were observed with each pulse discharge, no significant perioperative complications including pneumothorax, hemorrhage, infection, or cardiac arrhythmia were observed. Laboratory tests of the immediate postoperative group showed a significant decrease in serum albumin (*p* = 0.01) and a significant increase in lactic dehydrogenase (LDH, *p* = 0.024) and creatine kinase (CK, *p* = 0.033) (Table 1). Laboratory tests of the 2‐day postoperative group showed a significant decrease in hemoglobin (*p* = 0.006), but an increase in alanine transaminase (ALT, *p* < 0.001), aspartate transaminase (AST, *p* = 0.009), total bilirubin (*p* = 0.005), alkaline phosphatase (ALP, *p* = 0.001), and CK (*p* < 0.001) (Table 2). The laboratory tests of the 7‐day postoperative group still showed a slight decrease in erythrocytes (*p* = 0.001), hemoglobin (*p* = 0.006), and ALP (*p* < 0.001), but other abnormal indicators such as liver function and myocardial enzyme levels returned to normal (Table 3). These results preliminarily verified the safety of ultrasound‐guided percutaneous H‐FIRE ablation in porcine livers in vivo.

**TABLE 1:** Changes in the serum markers before and after H‐FIRE ablation (immediate).

Indexes	Before	After	*p* value
Red blood cell (10^9^/L)	6.7 ± 0.4	6.7 ± 0.2	0.904
White blood cell (10^9^/L)	15.9 ± 7.2	11.8 ± 5.8	0.489
Hemoglobin	113.3 ± 14.0	112.0 ± 4.4	0.883
Platelet	320.7 ± 43.2	244.3 ± 60.1	0.149
Alanine transaminase (ALT, U/L)	57.1 ± 0.7	69.9 ± 17.3	0.268
Aspartate transaminase (AST, U/L)	55.8 ± 4.2	399.7 ± 233.9	0.063
Serum Albumin (ALB, g/L)	45.2 ± 0.9	40.0 ± 1.8	0.010
Glutamyl transpeptidase (γ‐GT, U/L)	68.3 ± 10.1	76.3 ± 20.6	0.581
Direct bilirubin (DBIL, umol/L)	7.5 ± 0.5	7.7 ± 0.5	0.712
Total bilirubin (TBIL, umol/L)	16.4 ± 3.9	17.1 ± 1.5	0.792
Alkaline phosphatase (ALP, U/L)	164.4 ± 73.9	222.4 ± 100.4	0.466
Uric acid (UA, μmol/L)	79.6 ± 4.6	85.5 ± 2.5	0.128
Lactic dehydrogenase (LDH, U/L)	539.1 ± 3.3	775.4 ± 115.4	0.024
Creatine Kinase (CK, U/L)	689.6 ± 337.2	1644.0 ± 390.1	0.033

**TABLE 2:** Changes in the serum markers before and after H‐FIRE ablation (2 days).

Indexes	Before	After	*p* value
Red blood cell (10^9^/L)	6.3 ± 0.3	6.1 ± 0.2	0.248
White blood cell (10^9^/L)	15.5 ± 0.2	18.9 ± 2.6	0.085
Hemoglobin	104.3 ± 0.6	100.3 ± 1.2	0.006
Platelet	296.3 ± 60.5	262.3 ± 30.5	0.434
Alanine transaminase (ALT, U/L)	41.8 ± 0.6	77.9 ± 0.7	<0.001
Aspartate transaminase (AST, U/L)	48.9 ± 15.6	165.3 ± 39.8	0.009
Serum albumin (ALB, g/L)	43.0 ± 0.6	39.8 ± 0.6	0.002
Glutamyl transpeptidase (γ‐GT, U/L)	51.6 ± 4.6	49.6 ± 4.8	0.640
Direct bilirubin (DBIL, umol/L)	6.2 ± 0.2	5.8 ± 0.5	0.225
Total bilirubin (TBIL, umol/L)	11.6 ± 0.6	8.5 ± 0.5	0.005
Alkaline phosphatase (ALP, U/L)	123.7 ± 4.7	150.8 ± 2.6	0.001
Uric acid (UA, umol/L)	67.2 ± 3.9	118.3 ± 62.1	0.227
Lactic dehydrogenase (LDH, U/L)	580.2 ± 163.7	728.8 ± 131.2	0.287
Creatine Kinase (CK, U/L)	426.7 ± 4.8	2624.3 ± 193.6	<0.001

**TABLE 3:** Changes in the serum markers before and after H‐FIRE ablation (7 days).

Indexes	Before	After	*p* value
Red blood cell (10^9^/L)	6.7 ± 0.1	5.9 ± 0.2	0.001
White blood cell (10^9^/L)	20.6 ± 2.1	34.6 ± 11.4	0.105
Hemoglobin	118.7 ± 3.1	105.3 ± 3.1	0.006
Platelet	383.7 ± 34.6	518.7 ± 66.5	0.036
Alanine transaminase (ALT, U/L)	46.5 ± 1.4	45.3 ± 4.2	0.668
Aspartate transaminase (AST, U/L)	48.1 ± 7.7	56.1 ± 2.7	0.164
Serum albumin (ALB, g/L)	47.0 ± 3.0	44.3 ± 1.5	0.232
Glutamyl transpeptidase (γ‐GT, U/L)	60.6 ± 2.0	77.2 ± 19.8	0.224
Direct bilirubin (DBIL, umol/L)	7.2 ± 0.5	6.8 ± 0.1	0.312
Total bilirubin (TBIL, umol/L)	15.4 ± 1.5	13.1 ± 0.1	0.054
Alkaline phosphatase (ALP, U/L)	121.5 ± 3.0	76.2 ± 5.0	<0.001
Uric acid (UA, umol/L)	72.9 ± 4.7	77.9 ± 9.9	0.471
Lactic dehydrogenase (LDH, U/L)	478.4 ± 14.2	510 ± 14.2	0.053
Creatine Kinase (CK, U/L)	428.1 ± 126.8	577.4 ± 72.3	0.151

### 3.2 Imaging

**FIGURE 2:**
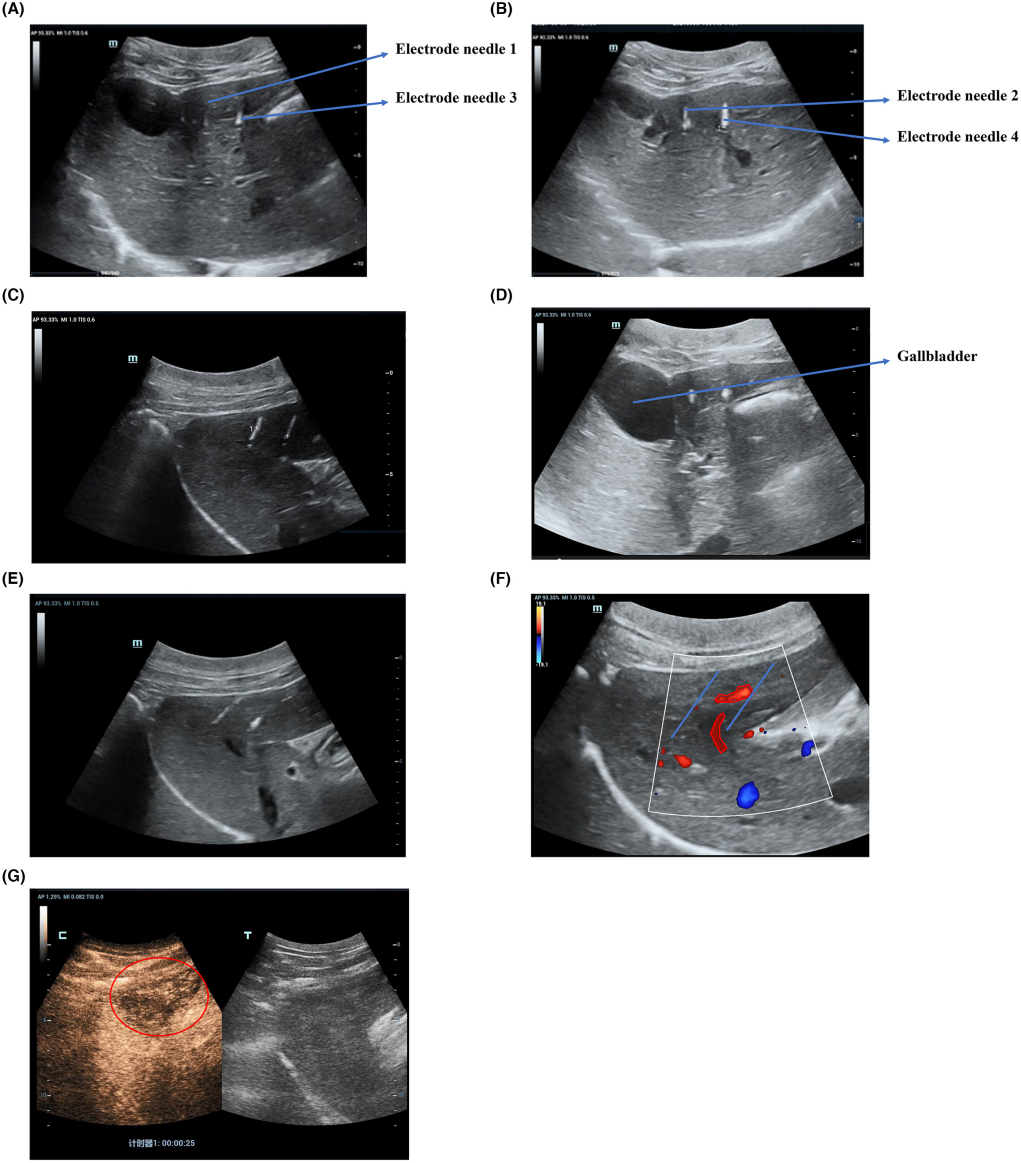
The liver close to the gallbladder was chosen as the target area for H‐FIRE ablation, and ultrasound was used to monitor the changes in ablation areas and ensure that the positions and depths of the four electrode needles did not change. (A) During the ablation process, ultrasound images showed that the gallbladder wall was in contact and electrode needle 1 was parallel to electrode needle 3. (B) Electrode needle 2 was parallel to electrode needle 4. (C) During the ablation process, all four electrode needles were parallel and 1.5–2.0 cm away from each other. (D) During the H‐FIRE procedure, the gallbladder wall is intact, and the bile ducts and blood vessels inside or adjacent to the ablation areas do not show any obvious echoic changes. (E) Ultrasonography performed immediately after H‐FIRE. On the ultrasound images, the ablation areas appeared isoechoic and only the needle track was clearly visible. (F) CDFI performed immediately after H‐FIRE showed that the blood flow of the vessels passing through the ablation region was normal. (The blue lines represented the electrode needles and the intact vessels were indicated by red circles). (G) CEUS examination performed immediately after H‐FIRE ablation showed that the ablation region was heterogeneously hypo‐enhanced with a blurry boundary. (The ablation area was indicated by a red circle).

We used ultrasound to monitor the changes in the depth and position of the four electrode needles and to ensure that there were no changes in the position or tissue injuries in the ablation areas. All four electrode needles were placed parallel to each other in the target ablation area (Figure 2A,B). During H‐FIRE, hyperechoic bubble streams were detected in the adjacent hepatic veins and ceased to appear after H‐FIRE stopped. Conventional ultrasound showed no obvious changes in the ablation area compared with the adjacent liver parenchyma. The gallbladder wall close to the ablation area was intact, and no obvious changes or injuries were observed in the bile ducts or blood vessels adjacent to the ablation area (Figure 2C,D).

Ultrasonography was performed immediately after the H‐FIRE. The ablation area appeared isoechoic without any acoustic shadows caused by air bubbles. However, the shape and margin of the ablation region cannot be clearly observed using conventional ultrasound. Only the needle track was clearly visible (Figure 2E). CDFI showed that the blood flow of the vessels passing through the ablation region was normal, and no significant changes were detected after H‐FIRE, indicating that the blood flow of the main vessels was not affected by H‐FIRE (Figure 2F). Furthermore, we performed CEUS immediately after the H‐FIRE procedure. CEUS showed that the ablation region was heterogeneously hypo‐enhanced with blurry boundaries during the arterial, portal venous, and delayed phases (Figure 2G).

**FIGURE 3:**
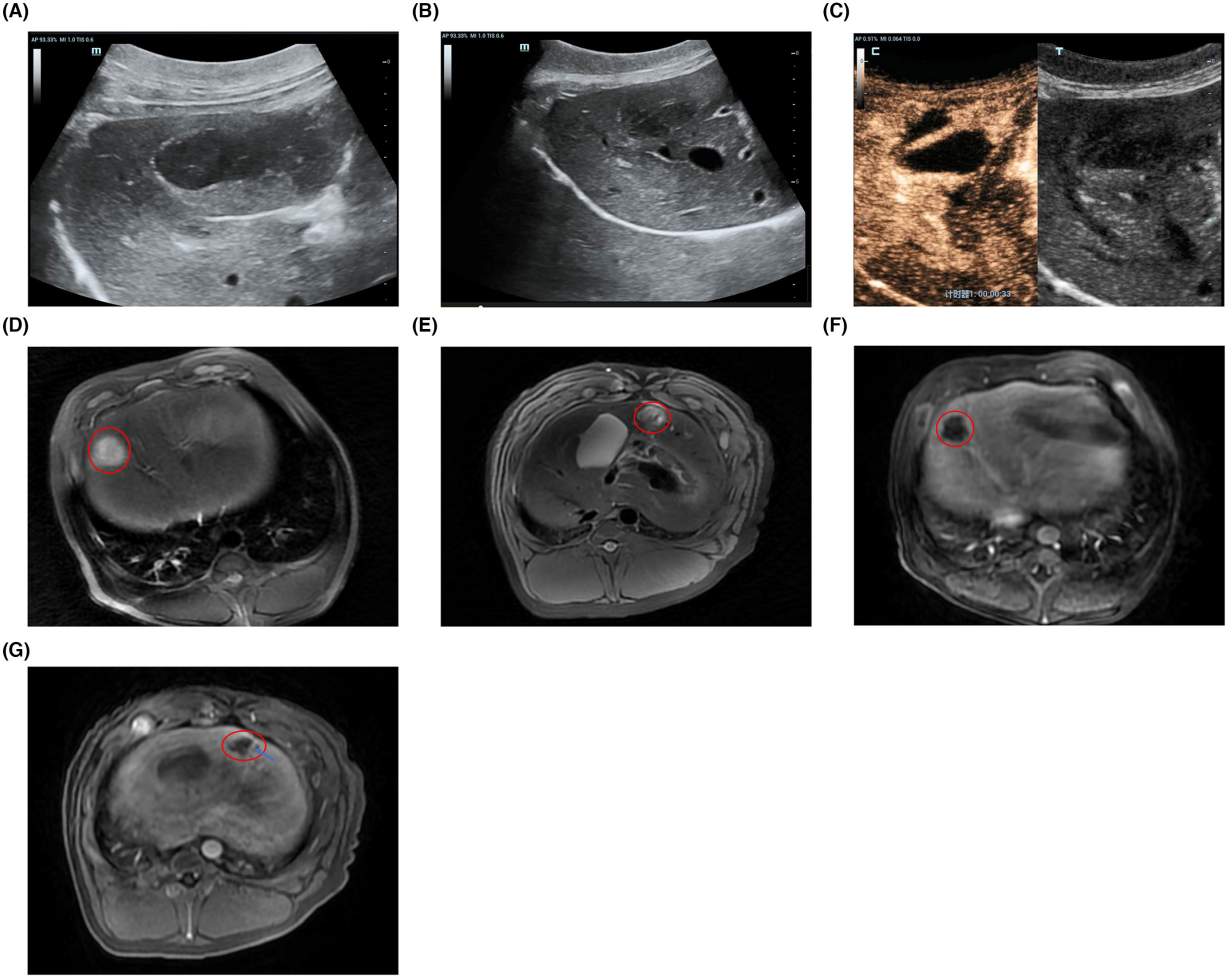
(A) Ultrasound was performed 2 days after H‐FIRE. On ultrasound images, the ablation area was hypoechoic, and the shape and margin were clearer. (B) The blood vessels passing through the ablation region were intact. (C) Two days after H‐FIRE, CEUS examination showed that the ablation region was completely non‐enhanced; however, the major blood vessels passing through this region still showed a normal enhancement pattern. (D) Contrast‐enhanced MRI was performed 2 days after H‐FIRE. The ablation areas appeared to be elliptical regions with high signal intensity on T2WI, and the boundary was clear. (E) The major blood vessel passing through the ablation area was intact on T2WI. (F) The ablation region showed no enhancement during all three phases. (G) The enhancement patterns of the major blood vessels passing through the ablation area were normal. (The ablation areas were indicated by red circles).

Two days after H‐FIRE ablation, ultrasound was performed to detect changes in the ablation areas. The ablation area was hypoechoic, and the shape and margin were clearer than 2 days ago (Figure 3A). Blood vessels passing through the ablation region were normal (Figure 3B). Subsequently, we performed CEUS and found that the ablation area was completely nonenhanced during the arterial, portal venous, and delayed phases. However, major blood vessels passing through this region exhibited a normal enhancement pattern (Figure 3C).

Dynamic contrast‐enhanced MRI was performed 2 days after H‐FIRE ablation. The ablation area appeared as an elliptical area of high signal intensity with clear boundaries on the T2WI (Figure 3D). The major blood vessels passing through the ablation area were intact (Figure 3E). The ablation region was not enhanced during the arterial, portal venous, or delayed phases (Figure 3F). But the enhancement patterns of major blood vessels passing through the non‐enhanced ablation areas were normal (Figure 3G).

**FIGURE 4:**
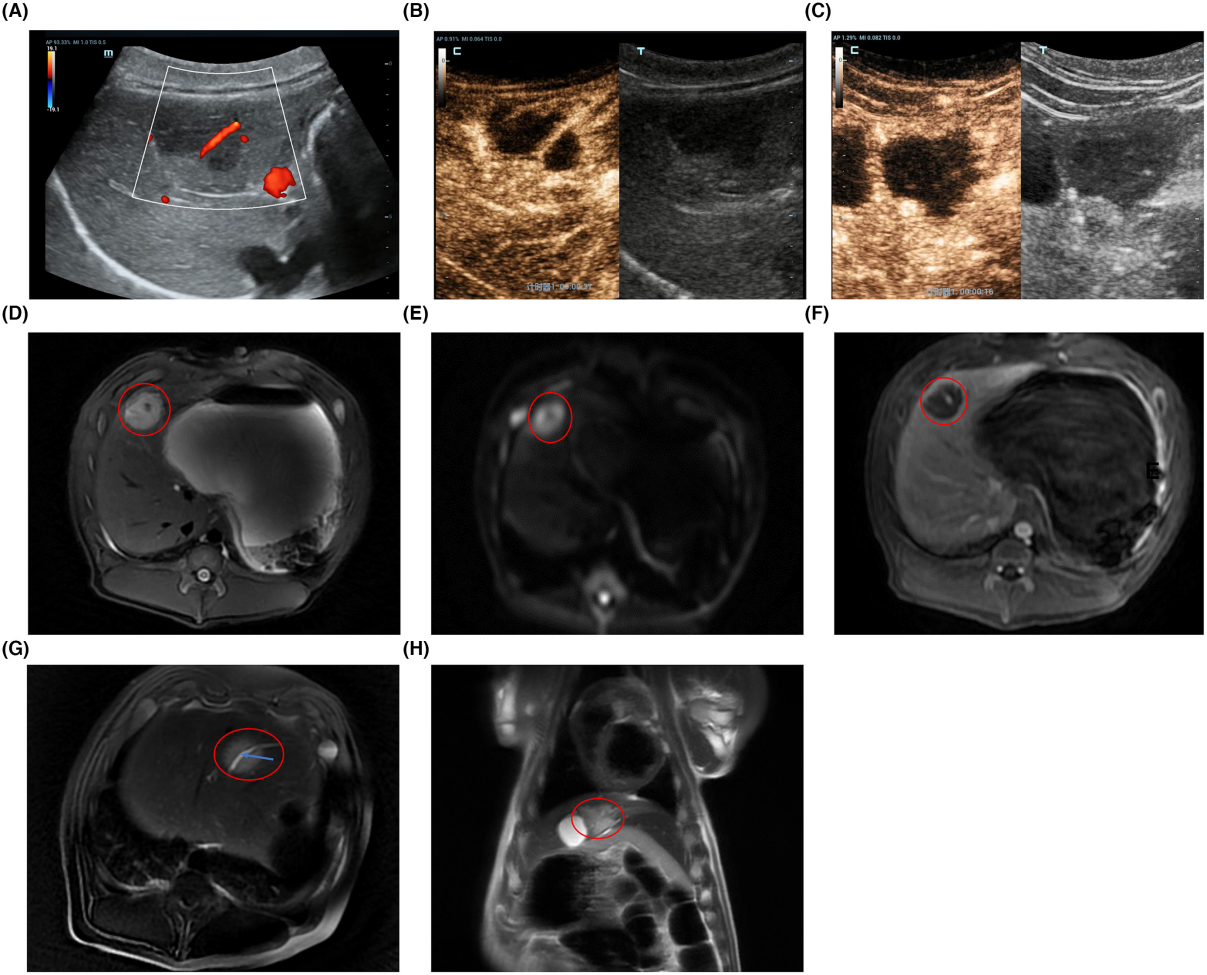
(A) Seven days after H‐FIRE, ultrasound showed that the ablation area appeared as a homogeneously hypoechoic region with a clear boundary. The blood flow in the vessels passing through the ablation region was normal. (B) The CEUS examination performed 7 days after H‐FIRE ablation showed that the ablation region was completely non‐enhanced, but the major blood vessels passing through this region still showed a normal enhancement pattern. (C) The CEUS examination performed 7 days after H‐FIRE ablation showed that the gallbladder wall was intact and the enhancement pattern was normal. (D) Dynamic contrast‐enhanced MRI was performed 7 days after H‐FIRE. The ablation areas still appeared to be an elliptical region with high signal intensity on T2WI, and the boundary was clear. (E) The ablation region was clearly visualized on DWI. (F) The ablation region showed no enhancement during all three phases. (G) The major blood vessel passing through the ablation area was intact. (The blood vessel in ablation area was indicated by a blue arrow). (H) The gallbladder wall adjacent to the ablation area was intact. (The ablation areas were indicated by red circles).

Seven days after the H‐FIRE procedure, ultrasonography was performed to detect changes in the ablation areas. The ablation area appeared as a homogeneously hypoechoic region with clear boundaries. The blood vessels passing through the ablation region were intact and CDFI showed normal blood flow (Figure 4A). Subsequent CEUS examination proved that, similar to the performance 2 days after H‐FIRE, the ablation area was completely non‐enhanced during the arterial, portal venous, and delayed phases. The enhancement patterns of the major blood vessels passing through this region are normal (Figure 4B). In pigs with ablation areas close to the gallbladder, CEUS revealed intact gallbladder walls and normal enhancement patterns (Figure 4C).

Dynamic contrast‐enhanced MRI was also performed 7 days after the H‐FIRE procedure. Similar to the performance 2 days after H‐FIRE, the ablation area still appeared as an elliptical area of high signal intensity with clear boundaries on the T2WI (Figure 4D). The ablation region was clearly visualized on diffusion‐weighted imaging (DWI) (Figure 4E). In the MRI dynamic enhancement sequence, the ablation region still showed no enhancement during the arterial, portal venous, and delayed phases. However, mild hyperenhancement was observed at the peripheral edge of the ablation area, which may have been due to the presence of an inflammatory edema band around the necrotic area (Figure 4F). The major blood vessels passing through the non‐enhanced ablation areas were intact and the enhancement patterns were normal (Figure 4G). In pigs with ablation areas close to the gallbladder, the gallbladder walls were intact and the enhancement patterns were normal in the MRI images (Figure 4F).

### 3.3 Pathological analysis

**FIGURE 5:**
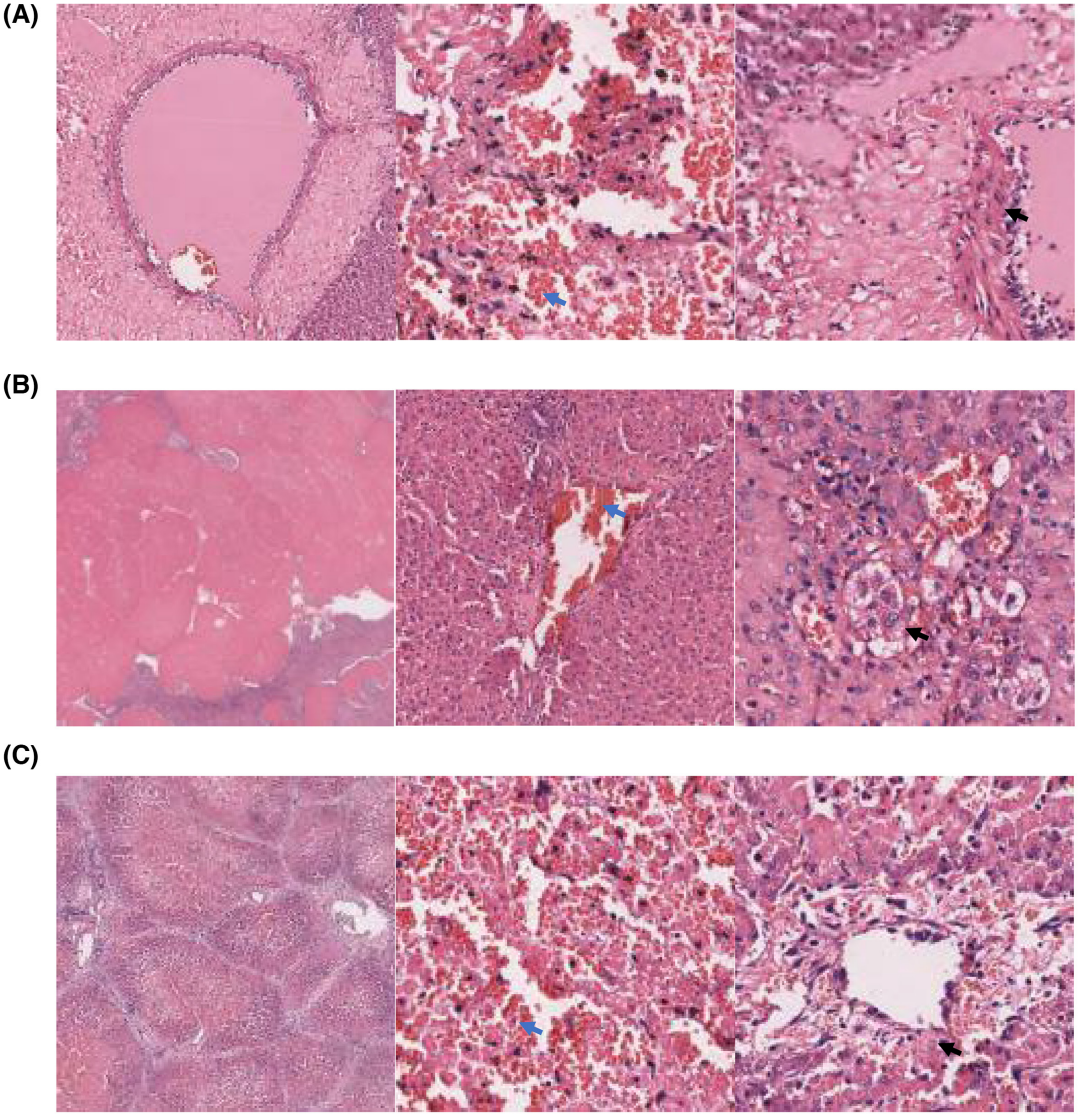
(A) The results of HE staining performed immediately after H‐FIRE ablation. (B) The results of HE staining performed at 2 days after H‐FIRE ablation. (C) The results of HE staining performed at 7 days after H‐FIRE ablation. The blue arrow referred to bleeding and necrosis, while the black arrow referred to inflammatory cells.

Immediately after H‐FIRE ablation, HE staining showed that the ablation areas could be clearly distinguished from the normal liver parenchyma with well‐defined boundaries. Multiple focal bleeding and necrosis were observed in the liver tissue. Fibrous tissue proliferation was observed in the interlobular septum with varying degrees of inflammatory cell infiltration. No damage was observed in the adjacent blood vessels or blood vessels within the necrotic tissue (Figure 5A).

Two days after H‐FIRE ablation, only focal hemorrhage and necrosis were observed under the membrane, with thickening of the membrane, proliferation of fibrous tissue, and significant infiltration of inflammatory cells. Partial liver tissue lesions presented as band‐like, patchy, or flocculent necrosis, with proliferation of fibrous tissue and small bile ducts and some infiltration of inflammatory cells. Normal lobular structures were present, and the portal area was enlarged, with fibrous tissue proliferation and significant infiltration of inflammatory cells (Figure 5B).

Seven days after H‐FIRE ablation, bleeding, and necrosis were observed in the liver tissue with a clear boundary from the surrounding normal liver tissue. Most small bile ducts and blood vessels within necrotic lesions were not damaged (Figure 5C).

These results revealed that ultrasound‐guided percutaneous H‐FIRE ablation using four electrode needles can induce complete necrosis of the target areas without destroying the small bile ducts or blood vessels within the necrotic areas.

## 4 DISCUSSION

High‐frequency irreversible electroporation is an improved IRE procedure and emerging alternative to thermal ablation (RFA and MWA).^26^ It could create more predictable ablations in the relative absence of the “heat sink effect” and treat benign and malignant tumors adjacent to large vessels and duct systems more effectively.^16^ Currently, CT‐guided minimally invasive ablation is considered a relatively mainstream imaging guidance strategy for abdominal surgery.^27^ However, its complex operation and radiation‐related side effects have limited its wide use. Ultrasound might be a more practical tool for percutaneous H‐FIRE ablation with the advantage of real‐time cross‐sectional scanning during the ablation process and closely monitoring the operation status.^28^ Our findings provide additional evidence that ultrasound‐guided percutaneous H‐FIRE ablation is technologically feasible and safe for porcine livers in vivo.

In our study, ultrasound‐guided percutaneous H‐FIRE ablations of experimental porcine livers near the gallbladder and vascular and biliary structures were successfully performed using four electrode needles. In line with previous studies, no significant perioperative complications such as pneumothorax, hemorrhage, or infection were observed in any of the pigs.^24^, ^29^ Blood test results showed that liver function indices and myocardial enzymes increased temporarily after H‐FIRE ablation but decreased to normal 7 days after ablation. The transient increase in myocardial enzymes might be caused by electrolyte disturbances affecting the myocardial blood supply during H‐FIRE ablation. The other serum indices were not statistically different before and after ablation at each follow‐up time point. Based on the physical properties of H‐FIRE, all electrode needles used for ablation need to remain parallel. Our study used conventional 2D ultrasound scanning to constantly monitor the changes in the depth and position of the four electrode needles and to ensure that there were no changes in position or tissue injuries in the ablation areas.

The heat generated by traditional RFA/MWA during the ablation process may damage the adjacent vascular and ductal structures, whereas emerging IRE and H‐FIRE ablations are largely restricted to the area within the electrodes and cause minor thermal damage.^13^ Because of the minor thermal effects in the ablation area, IRE and H‐FIRE could be performed close to the larger vessels and ducts without affecting the vascular walls and blood flow. Our CDFI findings demonstrated that the blood flow of the vessels passing through the ablation region was normal after H‐FIRE ablation using four electrode needles, indicating that H‐FIRE ablation did not affect the hemodynamic stability of the main blood vessels. Similarly, CEUS and dynamic contrast‐enhanced MRI revealed that the ablation region boundaries were well‐defined, and the gallbladder walls close to the ablation area were intact at the corresponding follow‐up time points. We also found that the main ductal structures passing through the non‐enhanced ablation areas were intact and the enhancement patterns were normal. The peripheral edge of the ablation area was mildly enhanced, possibly because of the presence of an inflammatory edema band around the necrotic area. Pathological analysis at different follow‐up time points after H‐FIRE ablation showed that the boundaries of the ablation areas were clear, and the vascular or bile duct structures in the ablation areas were preserved. These results indicate that H‐FIRE ablation was effective without damaging the surrounding or internal ductal structures and the gallbladder during treatment. Therefore, our study demonstrates the feasibility and safety of ultrasound‐guided percutaneous H‐FIRE ablation in porcine livers in vivo.

Previous studies and animal experiments have shown that the effective ablation area obtained using the dual‐electrode needle method frequently presented a spindle shape with a certain pulse time and voltage.^29^ However, in clinical practice, the ablation area might not reach the theoretical shape because of heterogeneous tissue electrical properties. In addition, the electrode needles need to be parallel and 1.5–2.0 cm away from each other in order to generate an effective electric field, it was challenging to arrange four needles under ultrasound‐guidance in clinical application. In the present study, all four electrode needles were smoothly implanted under ultrasound monitoring to avoid damage to the surrounding organs and blood vessels. Our study proposed and verified a four‐needle method for ultrasound‐guided percutaneous H‐FIRE ablation. This could ensure an effective ablation area and a sufficiently safe ablation boundary to cover the tumor. Notably, in the use of ultrasound guidance, ensuring the depth and parallel state depth of each needle through multi‐section scanning is the key to complete the accurate ablations. Moreover, a special attention should be paid to the physical damage caused by electrode needle insertion in areas close to the gallbladder or main ductal structure.

There were also some limitations in this study. First, because of the difficulties in establishing tumor models in large animals such as pigs, the present experiment only preliminarily verified the feasibility and safety of H‐FIRE ablation using four electrode needles in porcine livers in vivo; however, the effectiveness of this procedure in tumor ablation needs to be confirmed in future clinical trials, and its comparison with IRE and thermal ablation (RFA/MWA) in patients with liver tumors needs to be conducted. Second, our findings demonstrated that the ultrasound‐guided percutaneous H‐FIRE ablation technique using four electrode needles was feasible and safe, but the increased operative time and complexity associated with placing multiple electrode needles deserves further optimization.

## 5 CONCLUSIONS

In summary, this study demonstrated that ultrasound‐guided percutaneous H‐FIRE ablation in porcine livers in vivo was technically safe and feasible, and four‐needle method could be considered as a novel therapeutic option for the tumors in challenging locations of livers. This would help optimize the clinical application of H‐FIRE ablation.

## AUTHOR CONTRIBUTIONS

**Chang‐Tian Li:** Conceptualization (equal); data curation (equal); methodology (equal); writing – original draft (equal). **Guo‐Dong Zhao:** Conceptualization (equal); data curation (equal); formal analysis (equal); investigation (equal); validation (equal); writing – original draft (equal). **Wen‐Bo Zou:** Investigation (equal); methodology (equal); resources (equal); software (equal); supervision (equal); validation (equal); writing – original draft (equal). **Zhen‐Hua Zhang:** Investigation (equal); project administration (equal); resources (equal); software (equal); validation (equal); visualization (equal). **Yi Zhao:** Data curation (equal); investigation (equal); resources (equal); software (equal); visualization (equal). **Rong Liu:** Conceptualization (lead); funding acquisition (lead); project administration (lead); supervision (lead); writing – review and editing (lead).

## FUNDING INFORMATION

Not applicable.

## CONFLICT OF INTEREST STATEMENT

Dr. Chang‐Tian Li, Guo‐Dong Zhao, Wen‐Bo Zou, Zhen‐Hua Zhang, Yi Zhao and Rong Liu have no conflicts of interest or financial ties to disclose.

## ETHICS STATEMENT

This study was approved by the animal research and ethics committee of the Chinese PLA general Hospital (S2021‐407). All the procedures performed in this study were in accordance with the ethical standards of the institutional and/or national research committee and its later amendments or comparable ethical standards.

## Data Availability

The work dataset supporting the findings of this study is available upon reasonable request from the corresponding author, and the data are not publicly available because of privacy or ethical restrictions.
